# Method for multi-task learning fusion network traffic classification to address small sample labels

**DOI:** 10.1038/s41598-024-51933-8

**Published:** 2024-01-30

**Authors:** Lan Liu, Yongjie Yu, Yafeng Wu, Zhanfa Hui, Jun Lin, Junhan Hu

**Affiliations:** 1 School of Electronic and Information Engineering, Guangdong Polytechnic Normal University, Guangzhou, 510655 Guangdong China; 2 Sun Yat-Sen University, Guangzhou, 510006 Guangdong China; 3 China Electronic Product Reliability and Environmental Testing Research Institute, Guangzhou, 511370 Guangdong China

**Keywords:** Mathematics and computing, Computer science

## Abstract

In the context of the proliferated evolution of network service types and the expeditious augmentation of network resource deployment, the requisition for copious labeled datasets to facilitate superior performance in traffic classification methods, particularly those hinging on deep learning, is imperative. Nonetheless, the procurement and annotation of such extensive datasets necessitate considerable temporal and human resource investments. In response to this predicament, this work introduces a methodology, termed MTEFU, leveraging a deep learning model-based multi-task learning algorithm, strategically designed to mitigate the reliance on substantial labeled training samples. Multiple classification tasks, encompassing duration, bandwidth size, and business traffic category, are incorporated, with a shared parameter strategy implemented amongst tasks to assure the transference of information across disparate tasks. Employing CNN, SAE, GRU, and LSTM as multi-task learning classification models, training validation and experimental testing were conducted on the QUIC dataset. A comparative analysis with single-task and ensemble learning methods reveals that, in the context of predicting network traffic types, the accuracy derived from the multi-task learning strategy, even with a mere 150 labeled samples, can emulate the 94.67% accuracy achieved through single-task learning with a fully labeled dataset of 6139 samples.

## Introduction

As internet technology rapidly evolves and the number of users continually expands, we are also facing many new challenges. Though the rollout of 5G cellular networks and wireless sensor networks has enhanced the ease of people’s lives, it has also escalated the complexity of network topology^1^. The growing network traffic has led to an increasing number of network operation failures. In the face of current severe network failures and information security issues, accurate classification and rapid identification of network traffic are crucial.The task of network traffic classification plays a pivotal role in orchestrating network resource allocation. It can help network technicians control traffic trends in real-time, maintain network security and performance, reduce the adverse effects of network fluctuations on users, decrease the potential risks of major security incidents caused by network failures, and improve the service quality of Internet Service Providers and the service experience of users.

As research on traffic classification technology continues to deepen, an increasing number of traffic classification methods have been put forward by researchers and implemented in practical scenarios, among which the most widely used are methods based on machine learning and deep learning^2^. In the realm of traffic classification, a multitude of deep neural network algorithms have demonstrated effective classification results. However, harnessing traditional supervised machine learning and deep learning techniques requires substantial data training to achieve a high-performance classifier^3^. The process of capturing and annotating such a large volume of data is a complex task, consuming significant time and labor resources.

In response to the problem of deep learning models requiring a substantial volume of tagged data in network traffic categorization, we proposed a multi-task learning fusion (MTEFU) algorithm to To tackle the reliance issues of network traffic categorization models on labeled samples and effectively reduce the demand for labeled data. We use Various forms of deep neural networks as the foundational models under the multi-task learning framework, and perform training validation and testing on the classic QUIC dataset.

The challenges and contributions of this paper include:

1. Need for extensive labeled data: traditional machine learning and deep learning models are widely used in network traffic classification tasks, but these methods typically require a large amount of labeled data. Labeling data is often the most challenging and time-consuming process in building a classifier.
2. Application of multi-task learning framework: to address the above challenge, the paper reimagines the network traffic classification task within a multi-task learning framework. In this framework, in addition to predicting traffic category, it also predicts bandwidth requirements and flow duration. The motivation for this approach is twofold: first, bandwidth requirements and duration are very useful in many applications, including routing, resource allocation, and QoS (Quality of Service) provisioning; second, these two values can be easily obtained from each flow without the need for manual labeling or capturing flows in a controlled and isolated environment.
3. Improving accuracy: research shows that by using a large amount of easily obtainable data samples for bandwidth and duration prediction tasks, along with only a few data samples for the traffic classification task, high accuracy can be achieved.
4. Reducing dependence on extensive labeled data: the proposed multi-task learning framework reduces the need for a large labeled traffic dataset.
5. Experimental validation: the paper conducts two experiments using the QUIC public dataset, demonstrating the efficacy of the proposed method.

The structure of the subsequent sections of this paper is as follows. “Related works” primarily presents the related studies of deep learning in the field of network traffic classification research. “Method” introduces our suggested Multi-Task Learning Fusion (MTEFU) algorithm based on deep learning, input feature analysis, and source task classification partitioning method. “Experiment” conducts analysis and experiments on the benchmark dataset. “Discussion” explores potential issues and challenges. Lastly, “Conclusion” provides the concluding remarks.

## Related works

### Network traffic classification

While delving into the sphere of network traffic categorization, bolstered by machine learning and deep learning, academics predominantly apply methodologies such as supervised learning^4,5^, unsupervised learning^6,7^, and collective learning approaches. ^8,9^to distinguish the types of business traffic, such as email, web browsing, video streaming, etc.

Currently, many machine learning-based approaches for traffic classification have been suggested. Auld et al.^10^ A trained probabilistic neural network capable of classifying widely recognized peer-to-peer (P2P) protocols has been developed. including Kazaa, BitTorrent, GnuTella, etc., achieving an accuracy of 99%. Moore et al.^11^ employed a Naive Bayes classifier coupled with a Kernel Density Estimator to categorize based on the statistical attributes of the flow data. These features include the average, variance, median, quantile of packet size within the flow, and the time interval between packets, eventually achieving a classification accuracy of 96%. Draper et al.^12^ utilized the k-NN and C4.5 decision tree methodologies, characterizing network traffic using time-oriented attributes like the greatest and smallest arrival time gaps of packets. and ultimately achieved a classification recall rate of up to 92%. Using the C4.5 algorithm on a VPN dataset also achieved a recall rate of about 88%. Yamansavascilar and others^13^ manually selected 111 flow features depicted in reference^14^ and employed the k-NN algorithm^15^for classifying 14 types of applications, achieving a 94% accuracy. However, when using the k-NN classifier for prediction, the algorithm’s running time is a factor to consider. In 2016, Taylor et al.^16^ introduced a classification method grounded on burst data streams, taking into account the bidirectional nature of data stream transmission (interchanging source and destination addresses), independently tallying analyzing the sequence of packet sizes within the stream, computing 18 statistical elements encompassing aspects like mean, smallest value, largest value, quartiles, and so forth,for each sequence, and ultimately attaining a classification accuracy of 99% using the support vector regression methodology^17^and the random forest technique^18^. In 2019, Shen et al.^19^ put forward a method for recognizing decentralized applications, suggesting the use of kernel functions for merging features grounded on the statistical attributes of two-way data streams, further feature selection, and finally achieving a classification accuracy of 92%. The primary downside of traffic classification methods based on machine learning is the necessity for specialist knowledge to identify and sieve out features,rendering these methods both time-consuming and costly, and susceptible to human error. Consequently, scholars have begun to shift towards deep learning, which can autonomously learn features.

DeepPacket, a deep learning technique introduced by Lotfollahi et al.^20^, is premised on raw byte attributes of data packets. This approach treats each data packet as an individual input sample, negating the need for expert knowledge in feature extraction, and solely views the packet’s original bytes as features. The categorization model employs a one-dimensional convolutional neural network (1DCNN) and a sparse autoencoder (SAE), eventually reaching a classification precision of 98%. Wang and others^21^ proposed utilizing the initial 784 bytes of each data stream (either one-way or two-way) as input to the model and performed separate experiments on a one-dimensional convolutional neural network (1DCNN) and a two-dimensional convolutional neural network (2DCNN). The empirical results revealed that the 1DCNN outperformed, boasting an accuracy exceeding 90%. Li et al.^22^ harnessed recurrent neural networks (RNNs) for network traffic categorization and conceptualized a novel neural network - the Byte Segment Neural Network (BSNN). BSNN directly inputs data packets into the model and when five protocols, the mean F1-score of BSNN is roughly around 95.82%. Xie et al.^23^ put forth a flow categorization technique based on the self-attention mechanism known as SAM, which regards the raw bytes of each packet header as input to the model. This technique attained average F1-scores of 98.62% and 98.93% in discerning protocols and recognizing applications, respectively.

FS-Net, conceived by Liu et al.^24^, utilizes a deep learning approach centered around packet sequence characteristics within a flow. The time series characteristic harnessed in this approach is the size sequence of packets in the flow, and it introduces a reconstruction mechanism grounded on autoencoders. This mechanism equips the model to discern features that are most beneficial for classification and most emblematic of the data flow, eventually obtaining a classification precision of up to 99%. Lopez-Martin et al.^25^ introduced an approach that creates a $20\times 6$ matrix predicated on the port number, payload length, packet interval time, window size, etc., of the initial 20 packets of the data flow and feeds it into a combined model of convolutional neural networks (CNN) and long short-term memory recurrent neural networks (LSTM), with the ultimate accuracy surpassing 96%. Shapira et al.^26^ put forth an approach that morphs the data flow into images in line with the packet size and packet arrival time of the unidirectional data flow, and then classifies them via a CNN model, with the ultimate classification precision hitting 99.7%.

### Few-shot label learning

The above methods are all based on machine learning and deep learning for categorizing traffic. However, machine learning depends on the selection of statistical features, often requiring manual design of a set of feature sets that represent unique traffic characteristics, which requires a considerable degree of domain knowledge and a lot of practice. Deep learning methods have the ability to automatically extract features, avoiding complex feature selection work. Nonetheless, deep learning techniques frequently necessitate a significant amount of labeled datasets to demonstrate outstanding performance^27^. However, the collection and labeling of a large number of datasets require substantial time and exertion , and the dependence on a vast quantity of labeled samples limits the use of deep learning in many practical scenarios. To broaden the utilization contexts of deep learning techniques, researchers combine semi-supervised learning and deep neural networks, using the model transfer method in TL^28^ to reduce the requirement for substantial amounts of labelled datasets by deep learning models.

Rezaei et al.^29^ proposed a semi-supervised deep learning categorization technique that utilizes the characteristics of sampled data packets for traffic classification, and proved that the classifier trained by this method can enhance the precision of any task related to traffic classification. However, this method requires sampling the entire flow, which means that a large part of the flow needs to be observed before classifying the traffic, so it isn’t apt for real-time online categorization. Literature^30^ proposes a semi-supervised traffic categorization approach grounded on deep generative models. Instead of using manually made flow statistical features, this method uses a variational automatic encoder (VAE) to unsupervisedly extract latent features from the first 784 bytes of raw traffic information, and subsequently leverages a minor portion of labelled samples to train the traffic classifier of the deep generative model. When juxtaposed with the supervised strategy, this method only requires 20% of the labeled samples in the USTC anomaly detection dataset^31^, and the classifier can achieve an overall accuracy of over 95%. Aouedi et al.^32^ proffered a semi-supervised deep learning method based on stacked sparse autoencoder (SSAE). It first uses an unsupervised algorithm to pretrain SSAE, initializes parameters layer by layer, and then connects the supervised neural network classifier to the code layer of SSAE. It is fine-tuned using the backpropagation algorithm, and the influence of noise is avoided by injecting the hyperparameters of denoising codes. This approach is contrasted with various machine learning methods.

###  Comparison with existing research

In^33^, a general framework was proposed that provides direct guidance and direction for any traffic classification task. Most previous works fall under the overall framework. However, these methods depend on supervised learning and require a large amount of labeled data for training. This becomes even more challenging for deep models as they require more training data than classical machine learning methods. In the current survey, solutions to the demand for large labeled datasets are mainly divided into two categories, one of which is studied in^34^. This method includes a semi-supervised learning method, which first pre-trains the CNN model to predict a few statistical features in the sampled packets. They use the time-series features of sampled packets. Then, they replace the last few layers with new ones and retrain with a small labeled dataset. The advantage of their method is that it does not require manual labeling of the pre-trained dataset because statistical features can be easily calculated when the entire flow is available. However, their method requires sampling data packets, meaning that most of a flow needs to be observed before performing classification, which is not suitable for online applications. Secondly, and also the most recent work^35^, active learning (AL) as a subfield of ML, is a promising method to deal with the demand for a large number of labeled instances. The aim of AL is to reduce the demand for labeled examples by making intelligent queries for labels during the training process. The queries target examples that the AL algorithm believes will help construct the best model. Therefore, based on the challenges mentioned above, AL technology can be considered a suitable flow-based NTC technique, but it also has obvious disadvantages, sacrificing computational cost and the depth of the neural network framework to achieve its purpose, and has some limitations. In certain situations, labeling the most informative samples may not necessarily lead to the best performance of the model, especially when the data distribution changes rapidly. In this paper, we propose a multi-task learning method, overcoming the online network traffic classification of existing research, and not at the expense of its runtime and spatial complexity, and superior to single-task learning and ensemble learning classification processing.

## Method

To lessen the reliance of deep learning models on labeled samples, the multi-task ensemble fusion (MTEFU) algorithm is proposed. When faced with numerous interconnected tasks, multi-task learning assimilates these tasks simultaneously by utilizing general information across tasks and task-specific information. In this algorithm, we set three prognostic undertakings: bandwidth estimation, duration prediction, and traffic classification, with the first two as source tasks and the latter as the target task. This approach inherently shares certain parameters amongst tasks.The initial layers of the multi-task network share common information and assimilate different concluding layers to handle distinct outcomes. We use CNN, SAE, GRU, LSTM as multi-task learning classification models for training and evaluation.

**Figure 1:**
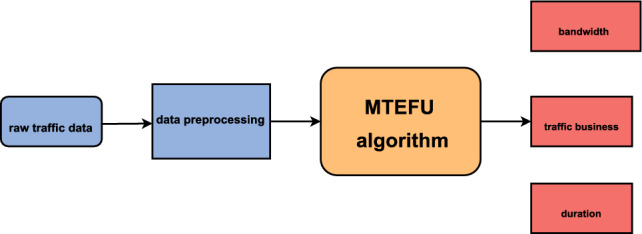
Overall framework for network traffic classification.

We propose an overall framework for network traffic classification As illustrated in Fig. 1, initially, we preprocess the original traffic dataset, including length discretization, length normalization, calculation of time intervals, and data interpolation. Then, we sequentially read the data and Segment it into training, validation, and test groups. Taking into account the previously mentioned time series characteristics, We designate the prediction of bandwidth and duration as supportive tasks, while setting the traffic type as our main goal. In the end, we employ the refined training set to cultivate the multi-task classification model, and the validation set is used to assess the model’s efficacy and tweak its hyperparameters. The algorithm for this framework is then tested on the test set.

### Principles of multi-task learning

Given a small sample of Dtrain, a simple model (such as a linear classifier) can be used to select a smaller H (hypothesis space). However, Network traffic categorization issues are generally intricate and cannot be adequately depicted by a small hypothesis h from a limited H. Therefore, it is best to use a sufficiently large H in a minimal amount of labeled samples, which makes the standard deep learning model infeasible. The multi-task learning method learns by limiting H to a more restricted hypothesis space H in line with preceding knowledge. In this way, The experimental risk minimizer’s reliability will increase, and the overfitting risk will be mitigated.$T_{1},\ldots ,T_{c}$,where some have few labeled samples and each task has a large number of unlabeled samples.

**Figure 2:**
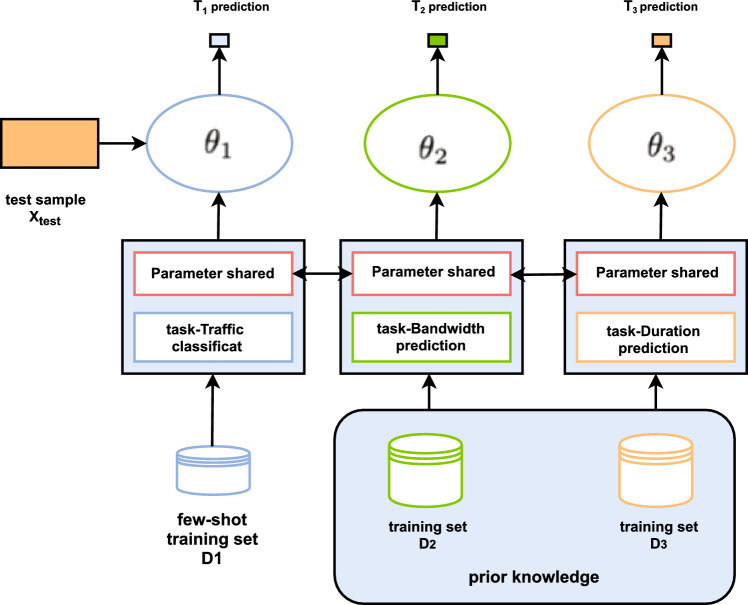
Schematic of the multi-task learning principle.

The schematic diagram in Fig. 2 illustrates the principle of multi-task learning. Each task Tc has a dataset $Dc=\{Dtrain,Dtest\}$, where the former refers to the training set while the latter pertains to the test set.Within these C tasks, we will divide the tasks that need to use a small number of labeled datasets as the target tasks, and the rest as source tasks.Multi-task Studying gains the parameters $\theta _{c}$ of $T_{c}$ from Dtrain. Given that these tasks are collectively learned, the parameters $\theta _{c}$ of $h_{c}$ gleaned for task $T_{c}$ are influenced by other tasks.

### MTEFU algorithm

Based on the manner of constraining task parameters, we characterize the approach within this schema as parameter interchangeable use. Traditional supervised learning methods require training on an abundant data set to generate a high-performing classifier. However, capturing and labeling a vast quantity of data is not easy, demands considerable time and labor, and the final classification accuracy is to be improved. A single learning method has difficulty learning the variances among them throughout the training procedure. Therefore, the proposed method performs three prediction tasks, and only the source tasks need manual and controlled environment training with small sample labeled data. Tasks related to forecasting bandwidth, duration, and traffic classifications. Regardless of whether data is obtained individually within a managed setting or otherwise, the overall bandwidth and length of each data stream can be effortlessly determined without manual labeling. By utilizing the rich data samples of Assignments concerning the forecast of bandwidth and duration, these rich data is able to be used to train the model’s shared parameters, thereby significantly improving the training process and accuracy of the source task traffic classification.

The MTEFU algorithm is meticulously designed to optimize the training of deep neural network models, focusing on precise network traffic classification. Through detailed input processing and output generation, the algorithm ensures efficient model training. At the input stage, the algorithm reads training, validation, and testing data from predetermined files, and undergoes a series of preprocessing steps (including data trimming, mask initialization, categorization, conversion of labels to one-hot encoding, and reshaping data) to prepare for the input to the deep neural network model. In the model definition phase, by constructing deep layers and adding dense layers, the model structure is meticulously set, and the use of categorical cross-entropy loss function and Adam optimizer in the compilation process further enhances the model’s performance.

During the training phase, the MTEFU algorithm skillfully combines training data, labels, and masks, ensuring comprehensive and in-depth model training. The validation phase uses validation data in real-time to ensure the quality and reliability of the model. Finally, the comprehensive testing in the evaluation phase not only verifies the model’s generalization ability across different datasets but also precisely outputs key performance indicators, such as accuracy. Overall, the MTEFU algorithm demonstrates its outstanding capabilities in data processing, model construction, and performance evaluation in the application of network traffic classification, fully embodying its efficiency and accuracy.

**Figure:**
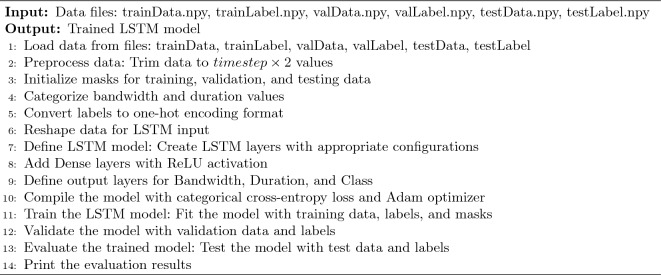
Algorithm 1 MTEFU algorithm.

### Preliminaries

In our multi-task learning framework, we leverage a variety of deep neural network classifiers for training, validation, and prediction. These classifiers include convolutional neural networks (CNN), sparse autoencoders (SAE), gated recurrent units (GRU), and long short-term memory (LSTM) networks, each playing a unique role within our model.

1. CNN: the foundation for feature extraction
  First proposed by Yann LeCun and others in 1989 , CNN is a powerful supervised learning algorithm based on hierarchical neural networks and trained using backpropagation. It independently identifies relevant attributes from source data through its convolutional and pooling layers, forming the basis for feature extraction.
2. SAE: a tool for unearthing deep features
  The Sparse Autoencoder(SAE) , a unique unsupervised learning algorithm proposed by Andrew Ng and others, generates new feature representations through autoencoding. It extracts complex and deep features from data by employing multi-layer stacking and sparsity constraints.
3. GRU: analyzing sequential data
  Introduced by Cho and others in 2014, GRU is a variant of Recurrent Neural Networks (RNN) with a gating mechanism to regulate information flow. It learns and memorizes long-term dependencies in sequence data, effectively handling tasks like natural language processing and time series prediction.
4. LSTM: complex dynamic memory structures
  LSTM, a distinct variety of RNN introduced by Hochreiter and Schmidhuber in 1997, incorporates mechanisms for ingress, forget, and egress, along with a crucial cell state. It captures long-term associations in sequences, excelling in processing sequence data and capturing long-term dependencies.

In summary, these deep neural network classifiers each fulfill their specific roles in our multi-task learning framework. They can either work together to form a robust system for sequence learning and feature extraction or be integrated into a single model, effectively supporting the processing of complex tasks like network traffic classification.

### Multi-task learning objective function

The tasks of predicting duration length, bandwidth size, and business traffic category are represented by B, D, and T, respectively. There are N training data, where $A_i$ denotes the input of the $i-th$data sample, and $y_i^B$,$y_i^D$ and $y_i^T$ represent the corresponding outputs for the tasks of predicting bandwidth, duration, and traffic type, respectively. The goal of parameter tuning in multi-task learning methods can be illustrated as follows:

1 $$\begin{aligned} \min (w) \frac{1}{2} \sum _{i=1}^N\left[ l\left\| y_i^B-A_i w_i\right\| ^2+l\left\| y_i^D-A_i w_i\right\| ^2+\lambda l\left\| y_i^T-A_i w_i\right\| ^2\right] +\rho \Vert W\Vert _{2,1} \end{aligned}$$

Here $w_i\in R^{n\times 1}$is equivalent to the weights in ordinary linear regression. $W=[w_1,w_2,\ldots ,w_k ]_{n\times k}$represents the weight matrix under multi-task learning, $\Vert W\Vert _2,_1=\sum _{i=1}^n\Vert w^i\Vert$.*l* represents the importance given to the loss function, $\rho$ is the regularization weight factor, used to reduce the model coefficients and the complexity of the model, preventing overfitting, $\lambda$ is set by the proportion of sample count in the bandwidth and duration tasks to the quantity of labelled samples in the traffic categorization task. Given that the training data samples for this task are significantly less than the other two auxiliary tasks, $\lambda$ can be marginally elevated to offset the shortage of labelled data. Pertaining to all training data, bandwidth and duration tags can be applied. Yet, only a minor fraction of the data samples bear traffic type labels.

### Input features

Within the domain of network traffic categorization, time series, headers, payload, and statistical attributes^33^ are frequently employed as the principal input characteristics. However, the use of header information has gradually been phased out due to its less-than-ideal accuracy. On the other hand, although statistical features provide comprehensive traffic information, they require complete traffic data, making them unsuitable for real-time prediction scenarios. Payload data has proven useful in certain specific datasets and types of communication^20,36^, especially when using unencrypted fields in the TLS1.2 handshake phase. However, with the emergence of new encryption protocols, such as QUIC and TLS1.3, these protocols minimize the number of unencrypted fields^29^, significantly reducing the usability of payload data. In our research, we propose a novel approach where we only observe the first few packets of traffic and then predict bandwidth and duration. The advantage of this method is that it can make predictions in real time, without having to wait for the completion of the entire stream. Therefore, we regard bandwidth and duration as distinct forecasting tasks rather than using them as input features as traditional traffic classification methods do. This not only improves the real-time nature of predictions, but also increases the accuracy of the predictions. The introduction of this method brings new ideas in the domain of network traffic categorization.

**Figure 3:**
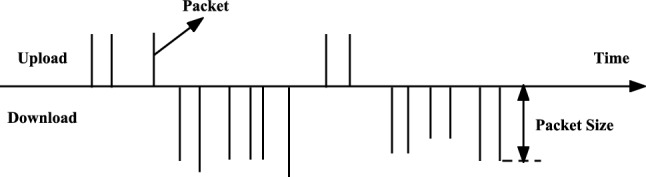
Schematic of data flow.

In our research,three time-series features from the initial k packets of network traffic were selected as inputs for our model. These three features are traffic identifiers, packet sizes, and inter-packet intervals. The model we invoke within the multitask learning framework takes as input a vector of length k, which contains information from two channels. The first channel contains traffic identifier information from the initial k packets, and the subsequent channel holds a combination of packet size and interval information. In our model, we consider packets as the basic units of network traffic. Thus, a network flow could be interpreted as an ordered series of packets. This depiction of the model is exhibited in Fig. 3. Within this figure^37^, each upright line symbolizes a packet, the elevation of the line portrays the packet’s size, and the space intervening between a pair of lines represents the temporal span separating two packets. The benefit of this methodology is that it can capture the dynamic nature of network traffic and can adapt to different network environments and traffic patterns. By using the information from the first k packets, our model can classify real-time network traffic, without having to wait for the completion of the entire flow.

In our research, we define bandwidth and duration labels as shown in Table 1, dividing bandwidth and duration into five categories, ranging from very low/short to very high/long. The fundamental aim of this categorization technique involves enhancing the understanding and classification of the bandwidth and duration traits of the QUIC dataset. Specifically, we first need to determine the threshold values for each category. These thresholds (A, B, C, D, W, X, Y, Z) can be set according to the characteristics of the QUIC dataset and practical requirements. For example, if the traffic in the QUIC dataset is mainly high-bandwidth traffic, we may need to raise the bandwidth thresholds (A, B, C, D). Similarly, if the traffic in the QUIC dataset is primarily short-duration traffic, we might need to lower the duration thresholds (W, X, Y, Z). Then, we can use these thresholds to assign categories to the bandwidth and duration of each packet. For instance, if a packet’s bandwidth is less than A Mbps, we will set its bandwidth category as “very low”. If a packet’s duration is between W and X seconds, we will set its duration category as “short”. This classification method has several major advantages. First, it can help us better understand and classify the bandwidth and duration characteristics of the QUIC dataset. By dividing bandwidth and duration into five categories, we can capture changes and patterns in network traffic more finely. Second, this method can enhance the effectiveness of multi-task learning. By setting more categories for each task, we can enable the model to learn more information, thereby improving prediction accuracy. Lastly, this method can also improve the model’s generalization ability. By using more categories, we can make the model better adapt to different network environments and traffic patterns.

**Table 1:** Bandwidth and duration class definitions.

Category	Bandwidth (Mbps)	Duration (s)
Very low/short	< A	< W
Low/short	A-B	W-X
Medium	B-C	X-Y
High/long	C-D	Y-Z
Very high/long	> D	> Z

## Experiment

In the experiment, deep neural network structures are used as base models in the multi-task learning framework. including (CNN, SAE, GRU, and LSTM) These were compared with single-task learning and ensemble learning.

### Implementation details

The proposed method uses the Python 3.9.5 Coding language and the Keras library 2.11.0 deep learning platform, along with the related experiments are conducted on the Microsoft Windows 11 operating system. All experimental algorithms are calculated using CPU, with acceleration by GPU. The specific Table 2 displays the parameters.

**Table 2:** Development environment.

Project	Properties
Operating system	Microsoft Windows 11
CPU	12th Gen Intel Core i9-12900H
GPU	NVIDIA GeForce RTX 3060 Laptop
Memory	32 GiB
Disk	1024 GiB
Framework	Python 3.9.5 + Keras 2.11.0

Our team undertook experimental procedures using the QUIC dataset. With the aim of avoiding overfitting of the model and maintaining optimal performance throughout the training phase, early stopping and model checkpoint methods were incorporated during the modeling process of the multi-task ensemble fusion unit (MTEFU) algorithm. We employ the GPU for enhanced speed, thus we selected a more substantial batch size and proportionately raised the epoch count. The model’s training was carried out through batch optimization utilizing the ADAM optimizer. By scrutinizing the alterations in accuracy and loss throughout the training stage, we implemented a suitable learning rate and incorporated Dropout into the neural network strata, providing an additional safeguard against overfitting.

### Benchmark dataset

In the rapidly evolving digital realm of today, network traffic is becoming increasingly diverse and complex. With the rise of online services, especially those provided by tech giants like Google, understanding the characteristics and patterns of network traffic becomes crucial for a range of applications, spanning from optimizing service quality to security monitoring. The QUIC dataset encompasses key services from Google, offering a comprehensive representation of modern network traffic patterns. Analyzing and classifying this dataset not only provides deep insights into widely used services but also challenges researchers to develop robust algorithms capable of handling real-world traffic scenarios. Thus, choosing the QUIC dataset for this experiment ensures a practical, relevant, and challenging foundation for developing and validating the proposed multi-task learning approach for network traffic classification. A detailed description of this dataset will follow.

The QUIC dataset, collected by the University of California, Davis, plays a pivotal role in this research. As depicted in Table 3, it covers five principal services from Google: Google Docs, Google Drive, Google Music, YouTube, and Google Search. These services are not only extensively utilized by hundreds of millions of users worldwide but also exhibit distinct network traffic characteristics for each service, furnishing us with a rich and diversified research sample.

Key features of the dataset include:

1. Authenticity: the dataset is derived from genuine user network activities and is not simulated or synthesized.
2. Diversity: it encapsulates a variety of distinct network services, each boasting its unique traffic pattern.
3. Interactivity and complexity: for instance, traffic from Google Docs pertains to data transmission activities when users edit, share, and collaborate on documents, necessitating our model to handle data with high interactivity and complexity.

The significance of the dataset for this research encompasses:

1. Real-world application scenario: given the dataset’s origin from genuine user network actions, the research outcomes are more likely to gain validation in real-world applications.
2. Research depth: the dataset’s diversity enables a more profound exploration into traffic patterns across various network services, thus offering comprehensive insights.
3. Method validation: the authentic and intricate nature of the dataset provides a platform to test the efficacy and robustness of our proposed method across diverse scenarios.
  In summary, the selection of this dataset offers our research a pragmatic, intricate, and diverse setting, allowing for verification and optimization of our method from multiple vantage points.

**Table 3:** Description of QUIC characteristics.

Service	Traffic type	Number of samples
Google Doc	Editing and collaboration traffic	1251
Google Drive	File storage and sharing traffic	1664
Google Music	Music streaming traffic	622
YouTube	Video streaming traffic	1107
Google Search	Search traffic	1945

**Figure 4:**
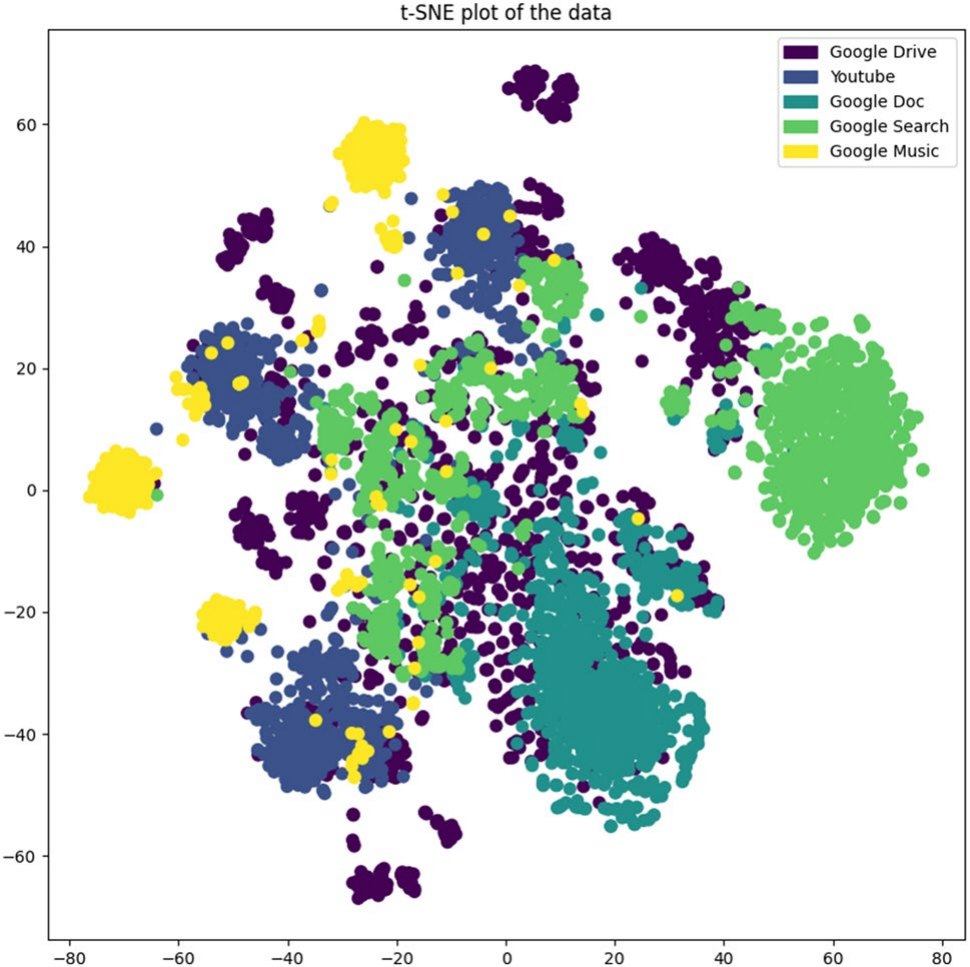
Visualization of the QUIC dataset using t-SNE.

We employed t-SNE for dimension reduction and QUIC visualization. t-SNE is an exceptionally efficient technique for dimensionality reduction, notably fitting for condensing data with a high number of dimensions are condensed to a 2 or 3-dimensional format for purposes of visual display. Throughout this procedure, data points with similarities are allocated to proximate locations in the condensed space, whereas data points with dissimilarities are designated to remote locations. Subsequently, the training labels were normalized, transforming the labels into values within the range of [0, 1]. This was done to represent different label categories in the subsequent scatter plot using colors. Finally, as shown in Fig. 4, a scatter plot was created using matplotlib, where each point represents a data point. Its location in the plot is determined by the result of t-SNE dimensionality reduction, and the color is determined by its corresponding label. From this scatter plot, we can observe the arrangement of data in space post-dimensionality condensation. Several key factors such as cluster clarity, degree of overlap, outliers, number and size of clusters can help us judge the difficulty of the dataset’s classification task. From the plot, it is observable that the extent of distinct categorizations of data points forming concise, dense groupings in the diagram is not particularly noticeable. Some data points overlap extensively in the plot, and there are some data points far away from any cluster (i.e., outliers). This indicates that classifying with single-task learning based on a minor volume of classified data is very challenging. Therefore, the application of a multi-task learning framework is very necessary to solve such network traffic classification problems.

### Data preprocessing

Algorithm 2 is a script for data processing. During the extraction of the dataset, owing to mistakes in extraction or inaccuracies in input, some data contains noise, duplicate values, missing values, and infinite values. Hence, our initial action is to conduct data preprocessing. The primary procedural steps are listed below.

Load and preproces data

**Figure:**
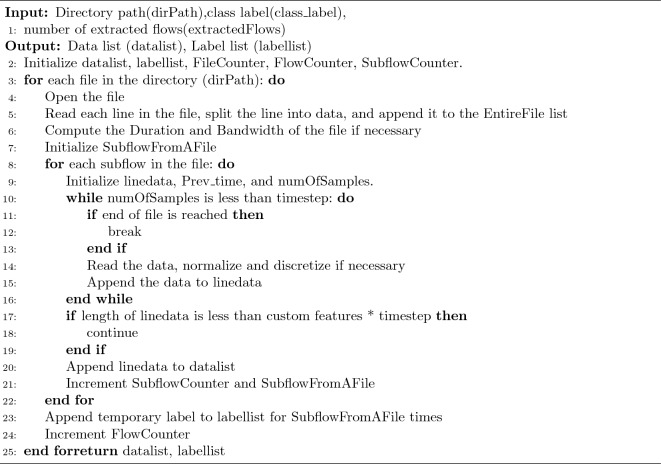
Algorithm 2.

1. Initialization stage: at this stage, some key data structures are first initialized, including lists for storing preprocessed data and corresponding labels, and counters for tracking file and stream processing progress. These data structures provide a basis for subsequent data processing and management.
2. File reading stage: at this stage, we begin to traverse all files in the specified directory. For each file, the code will open the file and read its contents. The contents of the file are split into data and added to a collection for subsequent processing.
3. Data preprocessing stage: at this stage, a series of preprocessing operations are performed on the read file contents. This includes calculating the file’s duration and bandwidth (if necessary), as well as discretizing and normalizing the data. These operations aim to boost the standard of the data and make the data more suitable for subsequent machine learning tasks.
4. Sub-stream processing stage: at this stage, each sub-stream in the file is processed. Firstly, the code initializes some variables, then reads the data under certain conditions, discretizes and normalizes the data, and adds the processed data to a list. This process helps us extract information useful for subsequent tasks and manage the size and complexity of the data.
5. Data storage stage: at this stage, if the processed data meets certain conditions, such as a length greater than or equal to a predetermined threshold, these data will be added to the data list, and the related counters will also be updated. In addition, the corresponding labels will be added to the label list.
6. Result return stage: at this stage, the data list and label list are returned. These two lists contain the preprocessed data and corresponding labels, respectively.

Especially important is the process of numerical normalization. To neutralize the interplay of dimensions amongst indicators, expedite gradient descent, and hasten model convergence, we normalize the data. Specifically, we employ the technique of computing Z-Score, resulting in each attribute having a mean of 0 and a standard deviation of 1, thereby transforming it into a standard normal distribution. This correlates with the overall sample distribution, and each sample point can impact the normalization. The normalization equation is presented below: Eq. (2) represents the mean of each attribute, Eq. (3) signifies the standard deviation of each attribute, and Eq. (4) corresponds to each column feature element.

2 $$\begin{aligned}{} & {} u=\sum _{i=1}^{N}x_{i} \end{aligned}$$

3 $$\begin{aligned}{} & {} \quad S=\sum _{i=1}^{N}(x_{i}-u)^2 \end{aligned}$$

4 $$\begin{aligned}{} & {} \chi _{i}^{'}=\frac{x_{i}-u}{s} \end{aligned}$$

### Evaluation metrics

**Figure 5:**
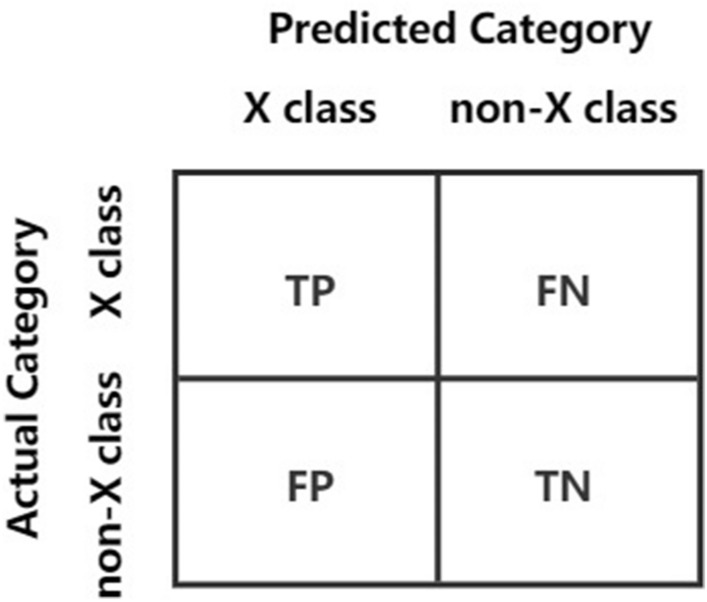
Binary classification confusion matrix.

The efficacy of network traffic categorization techniques can be evaluated using the confusion matrix. Figure 5 illustrates a straightforward binary classification confusion matrix^38^, where TP represents the probability that the actual sample is of class X and the predicted sample is also of class X; TN represents the probability that both the true category and the forecasted category by the classifier are not X; FP symbolizes the likelihood that the actual sample is not of class X and the predicted sample is of class X; FN symbolizes the likelihood that the actual sample is of class X and the predicted sample is not of class X.

Drawing from the aforementioned four notions, we can derive four distinct evaluative measures:

In Eq. (5), the ratio of the number of samples correctly predicted by the classifier relative to the overall count,

5 $$\begin{aligned} Accuracy=\frac{TP+TN}{TP+TN+FP+FN} \end{aligned}$$

In Eq. (6), among the samples predicted as class X by the classifier, the proportion of samples that are actually class X,

6 $$\begin{aligned} Precision=\frac{TP}{TP+FP} \end{aligned}$$

In Eq. (7), the proportion of instances classified as class X by the model compared to the actual quantity of instances that belong to class X,

7 $$\begin{aligned} Recall=\frac{TP+TN}{TP++TN+FP+FN} \end{aligned}$$

In Eq. (8), the weighted average of recall and precision.

8 $$\begin{aligned} F1\_Score=\frac{2\times Precision \times Recall}{Precision+Recall} \end{aligned}$$

Support: for each class, support the count of occurrences in the dataset that belong to that class.

Macro average: the macro average of each metric, which adds up the accuracy (or Recall / $F1\_Score$) of all classes and then divides by the number of classes, i.e., it does not consider the count of occurrences of each class.

Weighted average: the weighted average of each metric, which multiplies the accuracy (or Recall / $F1\_Score$) of each class by its support (i.e., the number of instances of that class), then sums it up, and finally divides by the aggregate count of instances.

### Experimental results

#### Introduction to the model structure

In our experiments, we first analyzed a comprehensive evaluation has been conducted on the performance efficacy of various architecture types within a training dataset manipulated through the multi-task learning fusion (MTEFU) algorithm. For each deep learning algorithm, the input shape is set to (120, 2), where the input shape is usually set to (timesteps, $input\_dim$), with timesteps being the time step length and $input\_dim$ being the quantity of attributes during each time stage. Throughout all stages of model training, we consistently utilize the Adam optimizer set at a learning rate of 0.001, running for a duration of 20 epochs with a batch size set at 64. The model’s blueprint and parameters are detailed in Table 4.

**Table 4:** Relevant structures and parameters of deep learning models.

CNN	SAE	GRU	LSTM
Input(120,2)	Input(120,2)	Input(120,2)	Input(120,2)
Conv1D (32 filters)	Flatten	GRU (32 units, return sequences)	LSTM (64 units)
Conv1D (32 filters)	Dense (128 units)	GRU (32 units, return sequences)	Dense (256 units)
MaxPooling1D	Dense (64 units)	GRU (128 units, return sequences)	Activation (ReLU)
Conv1D (64 filters)	Dense (32 units)	GRU (64 units, return sequences)	Dense (256 units)
Conv1D (64 filters)	Dense (64 units)	GRU (128 units, return sequences)	Activation (ReLU)
MaxPooling1D	Dense (128 units)	GRU (128 units)	Dense (5 units)
Conv1D (128 filters)	Dense (240 units)	Dense (256 units)	Dense (4 units)
Conv1D (128 filters)	Reshape	Activation (ReLU)	Multiply
MaxPooling1D	Flatten	Dense (256 units)	Dense (5 units)
Flatten	Dense (256 units)	Activation (ReLU)	
Dense (256 units)	Dense (256 units)	Dense (5 units)	
Activation (ReLU)	Dense (5 units)	Dense (4 units)	
Dense (256 units)	Dense (4 units)	Multiply	
Activation (ReLU)	Multiply	Dense (5 units)	
Dense (5 units)	Dense (5 units)		
Dense (4 units)			
Multiply			
Dense (5 units)			

#### Training and validation experiment

In this experiment, the dimension and scale of our training, validation, and testing datasets were key considerations. Specifically, our training dataset consists of 6139 samples, each with 240 features. The corresponding training label set contains 6139 samples, each with 3 target labels. Both the validation dataset and the testing dataset consist of 150 samples each, with each sample containing 240 features. The corresponding label sets each contain 150 samples, with each sample having 3 labels.On this basis of this dataset, we first conducted training validation experiments under the multi-task learning framework, divided into two sets of data. The first set used all 6139 unlabeled training data, each sample with 240 features. The second set used 100 labeled samples, each sample with 3 target labels, to train the entire model.

Regarding the model’s complexity, there’s a distinct differentiation between our proposed model and the baseline models. Firstly, from a spatial complexity perspective, our introduced model, due to its multi-task learning nature, typically demands more parameters for training, whereas the baseline models are relatively streamlined. However, our model, through parameter sharing and leveraging information from correlated tasks, can more efficiently utilize limited training data. Moreover, from a runtime viewpoint, although our model might necessitate a longer duration during training, owing to its optimization techniques and structural design, its inference speed in practical applications is comparable to, if not faster than, the baseline models.

**Figure 6:**
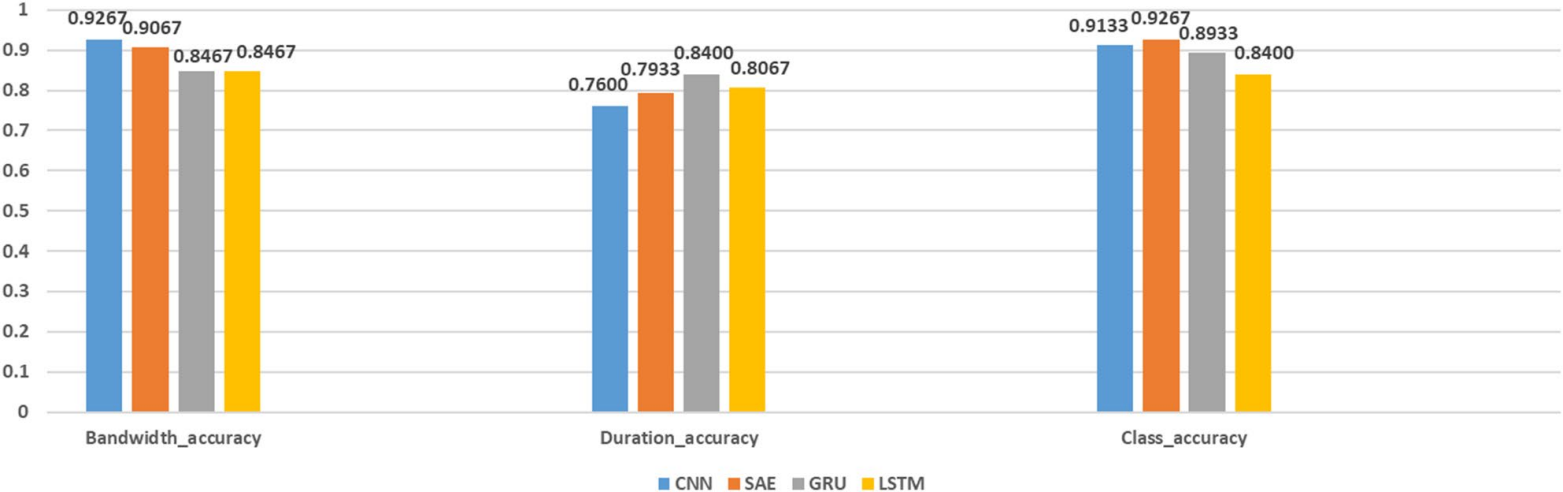
Comparing performance of different multi-task deep learning models.

As shown in Fig. 6, this set of data demonstrates the performance of four different deep learning models (CNN, SAE, GRU, LSTM) on three indicators. Overall, the CNN model showed the most balanced performance among all models, especially in terms of bandwidth accuracy, where the CNN reached the highest 92.67%, and also achieved 91.33% in classification accuracy. However, the duration accuracy was slightly inferior to other models, only reaching 76%, but the overall performance was excellent. However, for certain specific application scenarios, it may be necessary to focus on the optimal performance of a single indicator. For example, if classification accuracy is a concern, the SAE model achieved the highest 92.67%. This is related to its powerful feature extraction capability, Which can mine valuable insights from a substantial quantity of unlabeled feature data. If emphasis is placed on duration accuracy, then the GRU model performs best, with an accuracy of 84.00%. This is due to the model being a powerful sequence learning model.

**Figure 7:**
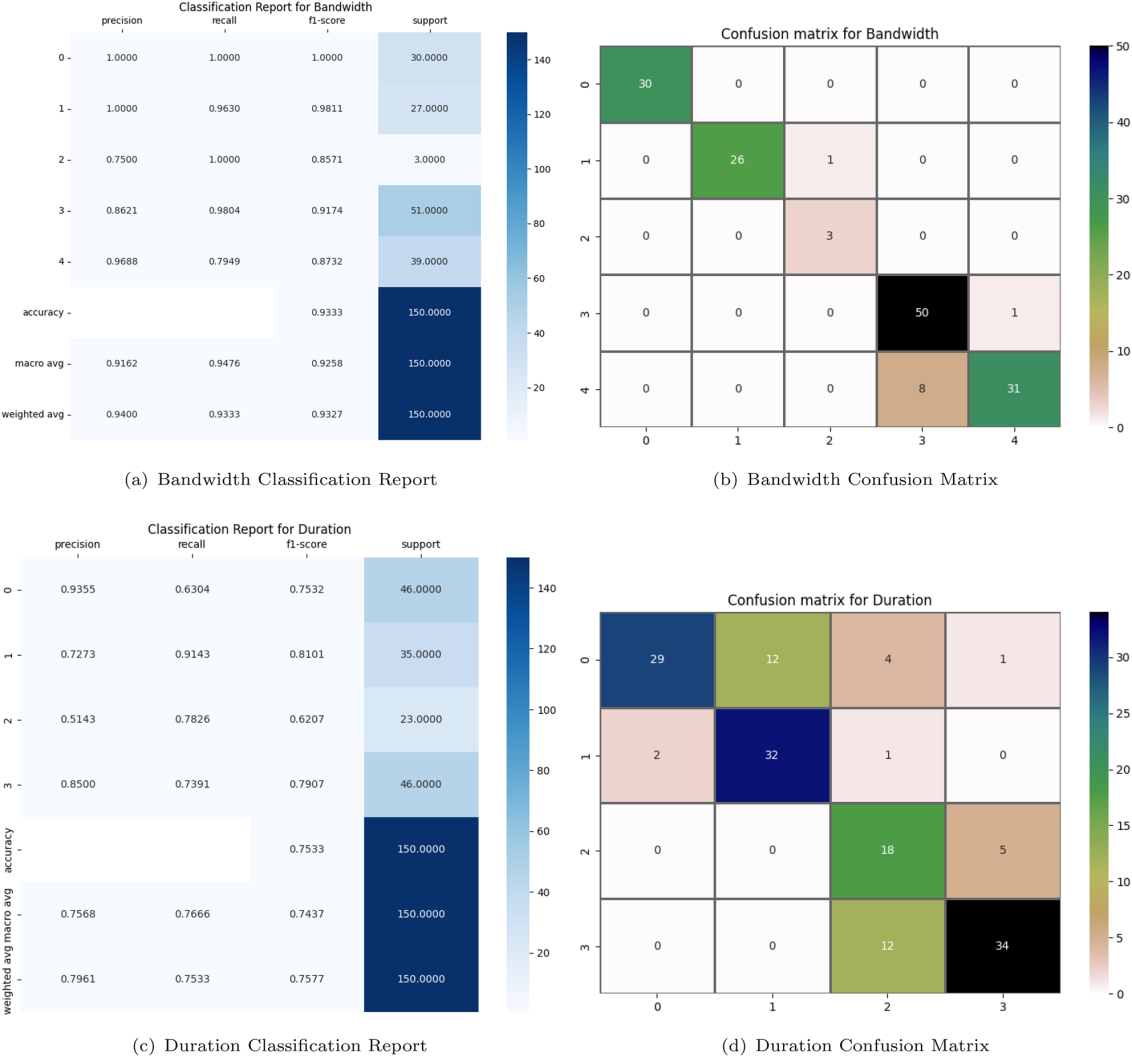
Source tasks for accuracy in QUIC dataset.

**Figure 8:**
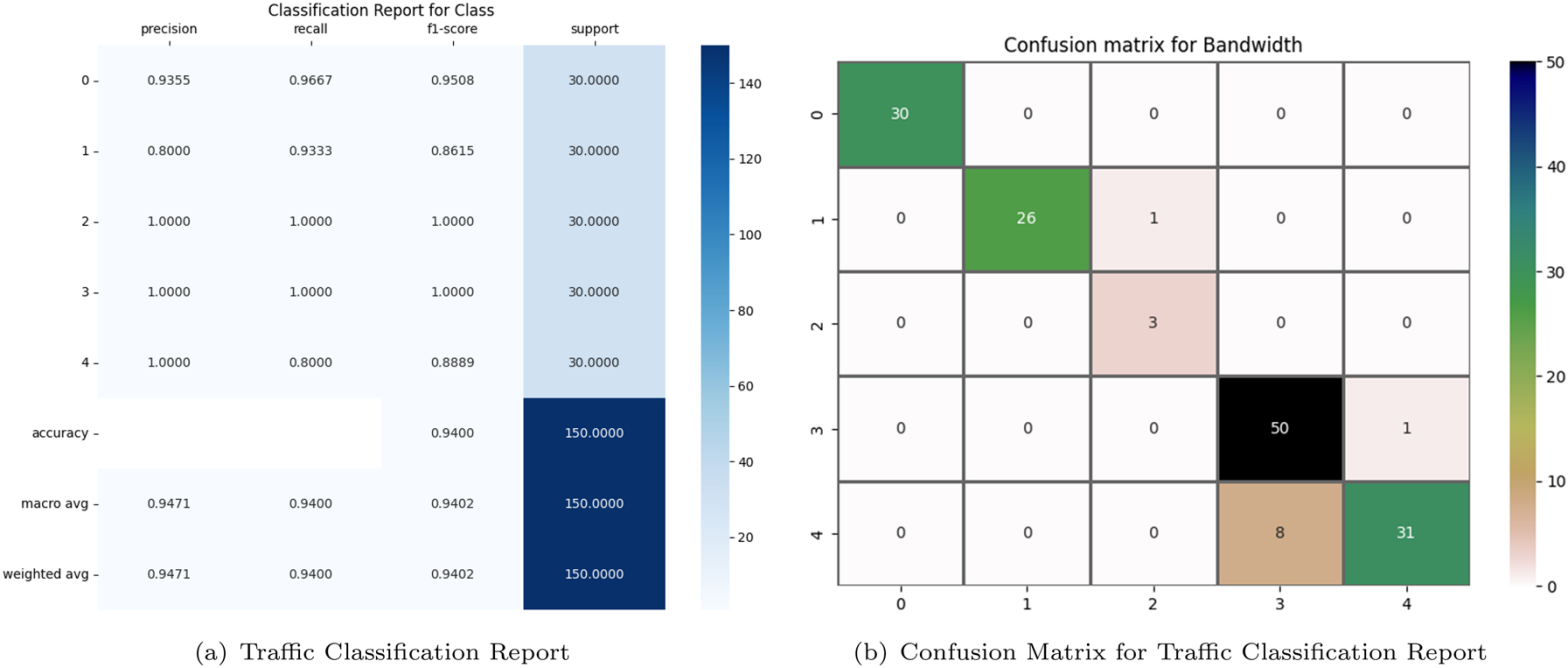
Target task for accuracy in QUIC dataset.

#### Comparison experiment on QUIC dataset

We also made predictions on the test data to generate predictive results. Comparing the predicted labels with the actual labels, we calculated and printed various performance metrics for each predicted category (in this case, ‘Bandwidth’, ‘Duration’, ‘Class’), including precision, recall, F1 score, and support for each category. These measurements provide detailed information on the model’s predictive performance, including its ability to correctly and incorrectly classify each category. Finally, we generated and visualized the confusion matrix, offering a more intuitive way to understand the model’s predictive performance, particularly the model’s misclassification in each category. Among the four different deep learning models, the CNN model demonstrated the most balanced overall performance, thus we carried out a comprehensive analysis on it.

As shown in Figs. 7 and 8,These six classification reports respectively display the model performance for “Bandwidth,” “Duration”, and “Class”. In the “Bandwidth” classification, the model shows strong performance, especially in dealing with category 0 (very Low) and 1 (Low), with an overall accuracy rate of up to 93.33%. However, for the classification of “Duration”, There’s a minor dip in the model’s performance, resulting in the accuracy percentage falling to 75.33%. Notably, the “Duration” category only has four groups. This might be because, during the data preprocessing phase, some “Duration” categories were merged or excluded, or it might be that only four different “Duration” categories were observed in the actual data collection process. Particularly in category 2 (Medium), the precision is only 51.43%, indicating challenges when dealing with data of certain specific categories. Nevertheless, for the classification of “Class”, the model’s performance is once again strong, with an overall accuracy rate of 94%. All categories have a precision of over 80%, with categories 2 (Google Music), 3 (YouTube) and 4 (Google Search) worthy of mention, as both precision and recall rates reached a perfect 100%. Overall, the model demonstrates superb performance in the classification tasks related to “Bandwidth” and “Class”, but needs further optimization for the “Duration” classification task, especially in dealing with the minority categories.

In this paper, our core research is to compare our multi-task learning strategy with single-task learning and ensemble learning strategies. In single-task learning, we selected two widely used models: random forest (RF) and convolutional neural networks (CNN), both of which possess statistical characteristics. For the ensemble learning strategy, we designed a model that combines convolutional neural networks (CNN) and recurrent neural networks (RNN) (CNN+RNN). For each task, we performed three model trainings from scratch. The entirety of the dataset was utilized in the training process for tasks involving bandwidth and duration prediction, because these tasks do not require manual labeling. This explains why the accuracy of the CNN+RNN model remains stable in predicting bandwidth and duration as the volume of labeled samples increases. RF and CNN models take the overall statistical characteristics of network flows as inputs because as long as the entire flow is available, bandwidth and duration can be obtained, which is why we did not train RF and CNN models in these pair of tasks.

As shown in Table 5, we found that single-task learning using a complete dataset containing all label categories could achieve an accuracy rate of 94.67%. However, in the task of predicting network traffic categories, the accuracy rate of the strategy of MTEFU Algorithm, when applied to a mere 150 labeled samples, results in an accuracy rate of 94.00%, which is comparable to the single-task learning strategy using a complete labeled dataset. This shows that the MTEFU Algorithm’s strategy, as evidenced, can significantly curtail the need for labeled data. In the task of predicting duration, the CNN+RNN model showed a significant advantage, with an accuracy rate of 92.00%. This is because the RNN algorithm can effectively extract time characteristics, allowing the ensemble learning method to surpass the multi-task learning method in predicting duration. However, its performance is slightly inferior to multi-task learning in predicting bandwidth.

**Table 5:** Accuracy on QUIC dataset.

Accuracy [bandwidth, duration, traffic class]				
Size of labeled samples	RF^36^	CNN^34^	CNN+RNN^25^	CNN-multi-task learning
10	[–,–,48.67%]	[–,–,85.33%]	[**89.33%**, **92.00%**, 64.67%]	[91.89%,74.00%,90.00%]
20	[–,–,64.00%]	[–,–,87.33%]	[**89.33%**, **92.00%**, 66.67%]	[92.89%,74.33%,91.00%]
50	[–,–,78.00%]	[–,–,88.83%]	[**89.33%**, **92.00%**, 76.67%]	[93.33%,74.33%,92.87%]
150	[–,–,**86.67%**]	[–,–,**90.00%**]	[**89.33%**, **92.00%**, 85.33%]	[93.33%,75.33%,**94.00%**]

Significant values are in [bold].

In summary, our research reveals that the performance of different learning strategies under deep learning models varies when handling different prediction tasks. When choosing a model, we need to determine the most appropriate one based on the specific task and the available number of labeled samples. In our case, the multi-task learning strategy (especially our CNN-Multi-task learning model) showed superior performance in all tasks and various labeled sample sizes, especially when the quantity of labeled samples is constrained.

## Discussion

1. In-depth comparative study on semi-supervised Learning
  In recent years, the application of semi-supervised learning in network traffic classification has been increasingly under the spotlight. Although it alleviates the pressure of extensive labeled sample requirements by maximizing the use of unlabeled data, selecting the appropriate source tasks remains a significant challenge. Future work is planned to focus on conducting comparative research on semi-supervised learning across various datasets, fully leveraging the advantages of deep learning models in attribute extraction and model training.
2. Challenges of class imbalance issues
  The issue of class imbalance in network traffic classification cannot be overlooked. When datasets are severely imbalanced among different categories, the model faces substantial challenges in classification prediction. Our future research will focus on developing and exploring different strategies to enhance the identification accuracy of imbalanced network traffic data, which will include strategies like over-sampling, under-sampling, or generating adversarial samples.
3. Selection and practicality of auxiliary tasks
  In the environment of multi-task learning, choosing suitable auxiliary tasks is crucial. Auxiliary tasks should not only be highly relevant to the main task but also have easily obtainable labels. Future work will concentrate on researching and analyzing various auxiliary tasks to find the optimal combination, improving the precision of traffic classification prediction, and considering it as a hyperparameter tuning problem.
4. Balancing between transfer learning and multi-task learning
  Within the context of network traffic classification, transfer learning and multi-task learning each bring unique challenges and opportunities. While multi-task learning demonstrates higher efficiency in certain aspects, transfer learning might be a more practical choice in some specific scenarios (such as those sensitive to computational complexity or with limited data labeling). Future research will delve into a deep exploration of the advantages and applicability of these two methods while also paying attention to the security issues triggered by using pre-trained models.

## Conclusion

As the internet technology evolves rapidly, the types of network services are increasing, making the network environment more complex. With the aim to offer high-caliber network services and enhance user satisfaction, accurate identification of various network services is crucial. However, the rapid emergence of new network applications means that traditional labeled samples cannot adapt to new classification tasks. Therefore, we need to find a way to effectively improve classification accuracy even when there is a decrease in the count of labeled samples.

For this purpose, we put forward a multi-task learning algorithm based on deep learning, called multi-task learning fusion (MTEFU). By excavating the optimal auxiliary task combination to enhance the precision of business category prediction, this process can be regarded as a kind of hyperparameter optimization method. We choose bandwidth and duration as source tasks, and business traffic classification as the target task. In addition, we also consider other prediction tasks as source tasks.

In our experiments, the accuracy of the multi-task learning strategy with only 150 labeled samples can rival the 94.67% accuracy of single-task learning using a complete labeled dataset of 6139 samples. The number of labeled samples for different task predictions varies, for instance, in the primary business network traffic prediction task, we labeled an average of 30 samples for each of the 5 main Google service categories in our QUIC dataset. However, there are slight variations in the auxiliary tasks, but the total number of labels remains constant. This shows that the MTEFU algorithm strategy can effectively reduce the demand for labeled data.

### Supplementary Information

Supplementary Information 1.

Supplementary Information 2.

## Data Availability

The datasets analysed during the current study are available in the https://drive.google.com/drive/folders/1Pvev0hJ82usPh6dWDlz7Lv8L6h3JpWhE.
