# Supplementary material for: Method for multi-task learning fusion network traffic classification to address small sample labels
Source: Sci Rep. 2024 Jan 30;14:2518. doi: 10.1038/s41598-024-51933-8 (PMC10827795; doi:10.1038/s41598-024-51933-8)
Supplement: Supplementary file 1 — Supplementary Information 1. [file 41598_2024_51933_MOESM1_ESM.zip › MultitaskTrafficClassification-master/.notebook/tmp/drawIOAppForChrome.html]

drawio
